# The impact of the World Health Organization 8-steps in wheelchair service provision in wheelchair users in a less resourced setting: a cohort study in Indonesia

**DOI:** 10.1186/s12913-016-1268-y

**Published:** 2016-01-22

**Authors:** Maria L. Toro, Chika Eke, Jonathan Pearlman

**Affiliations:** 1 Human Engineering Research Laboratories, VA Pittsburgh Healthcare System, Pittsburgh, PA USA; 2 Department of Rehabilitation Science and Technology, University of Pittsburgh, Pittsburgh, PA USA; 3 Programa de Ingenieria Biomedica, Escuela de Ingeniería de Antioquia y Universidad CES, Envigado, Antioquia, Colombia; 4 Department of Mechanical Engineering, Massachusetts Institute of Technology, Cambridge, MA USA

**Keywords:** Wheelchairs, Wheelchair service provision, Quality of life, Participation, Wheelchair skills, Less resourced settings

## Abstract

**Background:**

For people who have a mobility impairment, access to an appropriate wheelchair is an important step towards social inclusion and participation. The World Health Organization Guidelines for the Provision of Manual Wheelchairs in Less Resourced Settings emphasize the eight critical steps for appropriate wheelchair services, which include: referral, assessment, prescription, funding and ordering, product preparation,fitting and adjusting, user training, and follow-up and maintenance/repairs. The purpose of this study was to investigate how the provision of wheelchairs according to the World Health Organization’s service provision process by United Cerebral Palsy Wheels for Humanity in Indonesia affects wheelchair recipients compared to wait-listed controls.

**Methods:**

This study used a convenience sample (*N* = 344) of Children, Children with proxies, Adults, and Adults with proxies who were on a waiting list to receive a wheelchair as well as those who received one. Interviews were conducted at baseline and a 6 month follow-up to collect the following data: Demographics and wheelchair use questions, the World Health Organization Quality of Life-BREF, Functional Mobility Assessment, Craig Handicap Assessment Recording Technique Short Form. The Wheelchair Assessment Checklist and Wheelchair Skills Test Questionnaire were administered at follow up only.

**Results:**

167 participants were on the waiting list and 142 received a wheelchair. Physical health domain in the World Health Organization Quality of Life-BREF improved significantly for women who received a wheelchair (*p* = 0.044) and environmental health improved significantly for women and men who received a wheelchair as compared to those on the waiting list (*p* < 0.017). Satisfaction with the mobility device improved significantly for Adults with proxies and Children with proxies as compared to the waiting list (*p* < 0.022). Only 11 % of Adults who received a wheelchair reported being able to perform a “wheelie”. The condition of Roughrider wheelchairs was significantly better than the condition of kids wheelchairs for Children with proxies as measured by the Wheelchair Assessment Checklist (*p* = 0.019).

**Conclusions:**

Wheelchair provision according to World Health Organization’s 8-Steps in a less-resourced setting has a range of positive outcomes including increased satisfaction with the mobility device and better quality of life. Wheelchair provision service could be improved by providing more hours of wheelchair skills training. There is a need for outcome measures that are validated across cultures and languages.

## Background

For many persons with disabilities access to assistive technology (AT), such as wheelchairs, has been identified as a facilitator to full enjoyment of human rights [1–3]. Multiple studies in high income countries have concluded that access to wheelchairs is a vital component of rehabilitation and a determining factor in successful participation in society and employment [4–8]. Approximately 10 % of the world has a disability and 10 % of this section of the population requires a wheelchair because their ability to walk is limited [9]. Unfortunately, only 5 to 15 % of these individualshave access to an appropriate one [10]. Therefore, lack of access to appropriate AT has been a “missing bridge” to human rights and development especially in less resources settings [11–13]. Most users around the world rely on non-governmental organizations, charitable organizations, and other international organizations to access wheelchairs [14, 15]. International efforts to meet the needs started in the late 1970’s and early 1980’s. Ralf Hotchckiss, a wheelchair user in the United States (US), was a pioneer through Whirlwind Wheelchair by empowering wheelchair users in less resourced settings to build customizable wheelchairs that addressed local needs and incorporated locally available materials [16]. Motivation UK was foundedin the United Kingdom by David Constantine, a wheelchair user himself, as another method by which to meet the need for wheelchairs in less resourced settings [17]. Motivation UK placed emphasis on the clinical training and the service that needed to accompany the delivery of a wheelchair. In the early 2000’s large charitable organizations started mass-distributing wheelchairs [18]. Although this method of provision can reach many people in a relatively short period of time, the donations often do not meet criteria which ensure that each wheelchair will be more helpful to the user than harmful [19, 20]. Many of the donations consist of hospital-style wheelchairs designed for temporary use in institutional settings which do not meet international durability standards [21–24]. These wheelchairs often lack adjustability, are frequently provided without cushions, and typically do not meet the functional needs of users [22–25]. In addition, evidence suggests that these wheelchairs are frequently provided without associated services [22–25]. This means users’ needs are not assessed and not taken into consideration for the wheelchair selection [22, 23, 25]. When the wheelchair is delivered it is not fitted to the user and users are not trained in critical skills such as wheelchair mobility, maintenance, pressure ulcer prevention and proper transfer techniques [22, 23, 25]. Most of the anecdotal evidence has shown that the mass-distribution of wheelchairs without services has negative outcomes such as fatigue, discomfort, postural deformities and pressure ulcers which in the end lead to wheelchair abandonment [20, 21, 23, 26–29]. Conflicting anecdotal evidence suggests that a single-size hospital-style wheelchair provided to users who did not have one showed a decrease in number of pressure ulcers and improvement in participation at a 12 month follow up [30, 31]. In addition, high rates of wheelchair abandonment have been associated with poor device performance by not meeting nor withstanding the environment’s needs and selection of the device without consideration of user opinion [23, 28, 29]. Another criticism of this approach is that there is often no local capacity to repair the wheelchairs, including services, training and replacement parts. The user is left without a wheelchair once it is in state of disrepair [14, 23, 32–36]. In 2006, a consensus conference on wheelchairs for developing countries was held in India which brought a wide range of stakeholders involved in wheelchair provision in these settings [37]. The outcomes of this Conference placed the foundations for the World Health Organization (WHO) Guidelines for the Provision of Manual Wheelchairs in Less Resourced Settings (Guidelines) [10]. These Guidelines were used to motivate two Wheelchair Service Training Packages (WHO WSTP) that emphasize the eight critical steps for appropriate wheelchair services:referral and appointment, assessment, prescription, funding and ordering, product preparation, fitting and adjusting, user training, follow-up, maintenance and repairs. All steps push the effort to increase wheelchair service capacity in less resourced settings [38, 39] (Throughout the manuscript, we refer to this as WHO 8-Steps). The Guidelines and WHO WSTPs argue that in order to fully meet the needs of people with mobility impairments wheelchairs must be adjustable to fit the user, suitable for the user’s environment, available in the context where the user lives and accompanied by training in wheelchair use and maintenance [37, 40]. Furthermore, the Guidelines argue that a ‘perfect fitting’ wheelchair cannot solve the problem alone. It needs to be provided through comprehensive services which fully involve the users and their family [1, 10, 20, 25, 38, 39]. In addition, the United Nations Convention on the Rights of Persons with Disabilities (UNCRPD) was adopted in December 2006. Under the UNCRPD, independent mobility is a human right and people with disabilities are entitled to demand access to an appropriate wheelchair [11]. Although the Guidelines and the UNCRPD have been available for several years, the need for wheelchairs is still unmet; wheelchair services have yet to be fully implemented [24, 25, 30–32].

### Prior research in this field

Research-based outcome measures are scarce even though they are required to assess the impact of the service provision model. Previous methods of study involved obtaining self-reported data on health care, education, relationships, and AT performance [1, 21]. Positive effects of wheelchair provision include relief of caregiver burden [41], improved ratings of wheelchair propulsion, stability, transportability, and wheelchair skills [21]. It has been found that positive effects are dependent on proper fitting through trained local staff [21, 42–44]. Data has been collected through interviews of users and non-users of AT, and analyzed by comparing differences in outcomes between the two groups [1]. Many studies were cross-sectional [1, 15, 23, 25, 30, 45]; the three that were longitudinal studies did not compare to a control group [21, 24, 31] which reduces the reliability of their results. The strength of the evidence provided by these studies is limited. The situation in each less resourced setting is different and may need to be investigated individually [46, 47]. Research is needed to investigate the impact of different models of wheelchair provision that includes quality of life data at follow up and also includes children [3, 37]. Results will help guide national strategies to close the immense gap of access to appropriate wheelchairs in less resourced settings [1, 10, 18, 22, 36, 37, 48, 49]. To the best of our knowledge, no objective evidence is available regarding the impact of wheelchairs provided through the WHO 8-Steps of wheelchair service delivery; the goal of this paper was to gather objective data regarding the impact of these wheelchair services.

### Case-study of Indonesia

United Cerebral Palsy (UCP) Wheels for Humanity is one of the organizations working towards addressing the need for adequate wheelchair provision in areas with limited rehabilitation services with funding support through the United States Agency for International Development (USAID). They have established the organization called UCP Roda Untuk Kemanusiaan (UCPRUK) in Indonesia. UCPRUK works with volunteer seating specialists to provide appropriately fitted wheelchairs to people with limited mobility through the WHO 8-Steps [38, 50]. In Indonesia the Gross Domestic Product per capita is $3204, 8 % of the population lives on less than a $1 per day, and the mortality rate under the age of 5 per 1000 live births is 48 [51]. Around 20 % of the total 240 million population has a disability limiting day-to-day functioning and social activities [52]. Approximately 10 % of them, or 4.8 million people, require an appropriate wheelchair because their ability to walk is limited. Indonesia ratified the UNCRPD in 2011 which in-principle means the Indonesian government supports equal rights and opportunities for persons with disabilities [2]. Unfortunately, people with disabilities in Indonesia are at high risk for poverty and face social barriers leading to unproductivity and dependency [52]. Youth with disabilities are more likely to live in low income households and less likely to be in school than their peers without disabilities [51]. The government provides health insurance to those who are poor but it does not include assistive technology such as wheelchairs or prosthetic devices [52]. Limitations in appropriate provision of assistive devices include the lack of training in seating and mobility and the lack of coordination between providers to insure the best possible outcome through technology [53].

The purpose of this study was to investigate the impact of the UCP’s wheelchair provision services, which are provided according to WHO’s 8-step program. Specifically, the goal was to investigate how wheelchairs provided to individuals with mobility impairments related to mobility, participation in society, quality of life, wheelchair skills, wheelchair maintenance, and satisfaction with mobility as compared to a control group. The results of this study will help inform efforts to further develop the wheelchair provision process at UCPRUK and help guide the Indonesian government on appropriate wheelchair provision.

## Methods

### Ethical considerations

The Medical and Health Research Ethics Committee from the Faculty of Medicine at Gadjah Mada University (Yogyakarta, Indonesia) provided approval to conduct the study. Written informed consent from all participants was obtained before implementing study procedures. Where participants were children, written consent was provided by a parent/guardian on behalf of the child. Data for this study was collected between April 2013 and April 2014, by members of the UCP team. No incentives for participation were offered. The University of Pittsburgh (Pitt) Institutional Review Board approval for de-identified data transfer was obtained prior to data analysis. A team of Pitt researchers performed the analysis and have prepared this manuscript.

### Instrumentation

Demographic information such as date of birth, gender, presence of pressure ulcers, employment/education status, and nature of disability were collected from each participant. Having a wheelchair at baseline and number of hours of wheelchair use at follow up were collected. A series of questionnaires were used to interview subjects or proxies to evaluate functional mobility, participation in society, quality of life, wheelchair skills, and wheelchair maintenance:

1. Craig Handicap Assessment Recording Technique Short Form (CHART-SF): measures community involvement based on 6 domains: physical independence, cognitive independence, mobility, occupation, social integration, and economic self-sufficiency [54, 55]. In order to determine economic self-sufficiency, the procedure listed in the scoring form was adjusted by replacing the poverty level for the U.S. with the poverty level in Indonesia. Additionally, new codes were developed to process responses about income and medical expenses in rupiah instead of dollars.
2. World Health Organization Quality of Life-BREF (WHOQOL-BREF) Bahasa Indonesia version: “assesses the individual’s perceptions in the context of their culture and value systems, and their personal goals, standards and concerns” [56]. It contains physical health, psychological health, social relationships, and environment domains.
3. Wheelchair Skills Test Questionnaire (WST-Q): is a safe, valid and reliable method to objectively assess the skills of wheelchair users, such as their capability of putting on brakes, propelling a straight distance, and doing a reaching task from their wheelchair [57–59]. Adult and Child participants in the wheelchair group answered the user version which assesses their independent wheelchair skills. Proxies answered the caregiver version (31 items) for the Adults + proxy and Child + proxy groups. This version assessed the skills of the caregiver pushing the wheelchair with the user in it. A total Capacity score (what the person can do) and a total Performance score (what the person actually does) was calculated.
4. Wheelchair Assessment Checklist (WAC): is a screening procedure that consists of a checklist and scoring system for categorizing wheelchairs based on their physical and working conditions [60]. A total score was calculated based on the condition of all relevant components.
5. Functional Mobility Assessment (FMA): a self-report outcomes tool designed to measure effectiveness of mobility, such as whether the user can carry out their daily routine in an independent manner [61]. An adult and a child-adapted version were used [61, 62]. In the cases were the proxy answered, the answers were related to the wheelchair user’s experience.
6. Wheelchair technology: questions about wheelchair repairs and adverse consequences of these repairs were asked.

All questionnaires except the WHOQOL-BREF were translated from English to Bahasa Indonesia language. All responses were translated back into English by a professional translator and reviewed for errors.

Data collector training was done on-site at UCPRUK in Yogyakarta in Java, Indonesia by 2 personnel from the University of Pittsburgh before the participant recruitment and enrollment was started. One of the trainers was a Bahasa Indonesia native speaker. Five data collectors were trained on administering all questionnaires during a 5-day workshop. Following input from the training week, the translated questionnaires were finalized.

### Procedure

Participants who came to UCPRUK to receive services for a new wheelchair were invited to participate in the study. Inclusion criteria were people with mobility impairments coming to UCPRUK for a new wheelchair. Children and adults were invited to participate and caregivers were enrolled as ‘proxy’ subjects for those who could not self-propel. UCPRUK provides services on a first-come, first-served basis and the demand for wheelchairs is always larger than what they can provide immediately.

Participants are usually placed on a waiting list until wheelchairs and services are available. Depending on where the participant was on the waiting list, they were categorized as on the waiting list (Waitlist group) or intervention (Wheelchair group). Participants in the Wheelchair group received a physical and interview assessment to identify their needs. The most appropriate wheelchair from those available was selected and prescribed. Then, this new wheelchair was delivered and fitted to the participant. During the same appointment, he/she was trained on how to handle it, how to transfer, basic maintenance, and how to contact the UCPRUK if they had problems. The wheelchairs were donated by UCPRUK.

Table 1 describes the questionnaires that were administered for each type of participant at baseline and at follow up. Only participants in the wheelchair group were asked questions related to the wheelchair. Paper-based questionnaires were used during data collection.

**Table 1:** Questionnaires administered at baseline and follow up by type of participants and waitlist or wheelchair group

Questionnaire	Waitlist group								Wheelchair group							
	Baseline				Follow up				Baseline				Follow up			
	A	A + p	C	C + p	A	A + p	C	C + p	A	A + p	C	C + p	A	A + p	C	C + p
Demographics	✓	✓	✓	✓	✓	✓	✓	✓	✓	✓	✓	✓	✓	✓	✓	✓
WHOQOL-BREF	✓	✓	-	-	✓	✓	-	-	✓	✓	-	-	✓	✓	-	-
CHART	✓	✓	✓	✓	✓	✓	✓	✓	✓	✓	✓	✓	✓	✓	✓	✓
FMA adults	✓	✓	-	-	✓	✓	-	-	✓	✓	-	-	✓	✓		
FMA kids	-	-	✓	✓	-	-	✓	✓			✓	✓	-	-	✓	✓
WST-Q user	-	-	-		-		-		✓^a^		✓^a^		✓		✓	
WST-Q caregiver	-	-	-	-	-	-	-	-	-	✓^a^	-	✓^a^		✓		✓
WAC	-	-	-	-	-	-	-	-	-	-	-	-	✓	✓	✓	✓
WC repair/consequence	-	-	-	-	-	-	-	-	-	-	-	-	✓	✓	✓	✓

^a^Only administered if participants had a wheelchair a baseline

A: Adult, A + p: Adult + proxy, C: Child, C + p:Child + proxy

**Fig. 1:**
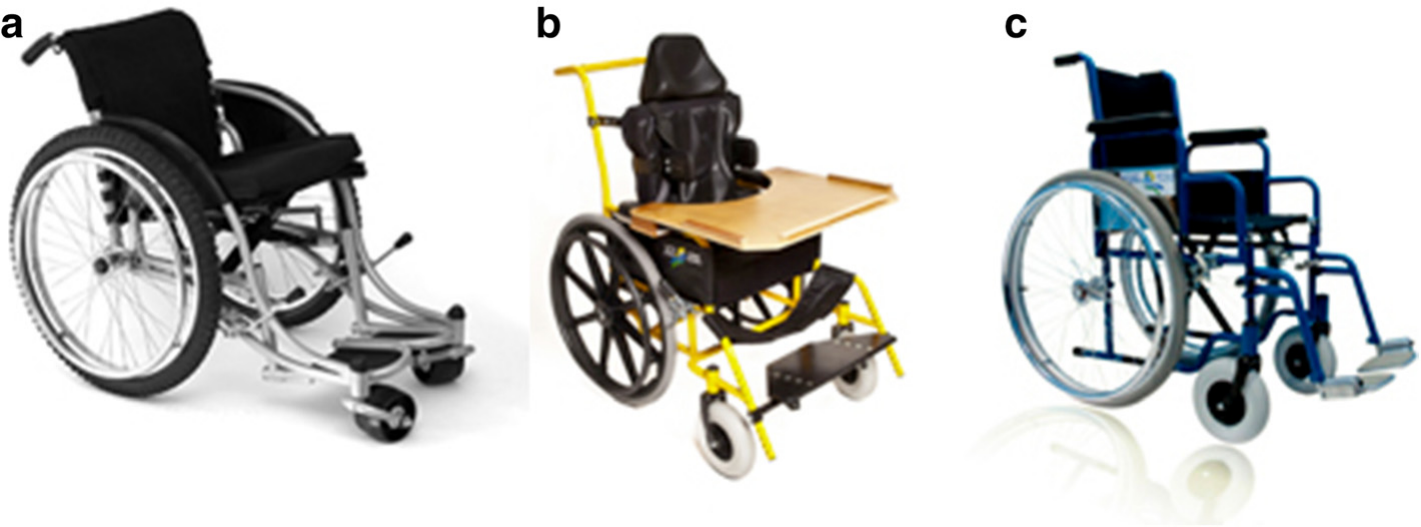
Types of wheelchairs available for provision at UCPRUK. **a** Roughrider **b** Kids **c** Harmony. Images are property of UCPRUK

Participants in the wheelchair group were assessed based on their needs and the most appropriate wheelchair out of four types was provided (pictures of the wheelchairs are shown in Fig. 1). The specialized wheelchair is not shown; it is a manual wheelchair with reclining back support.

### Data reduction and statistical analysis

#### Power calculation

The CHART was selected as the main outcome for the power analysis calculation because it is a commonly used participation measure in rehabilitation research. For a power of 80 %, alpha 0.05, and a mean of paired differences of 25 points we need 30 of each distinct type of participants. To plan for attrition, we had a recruitment goal of 40 participants for each participant type for the Wheelchair and Waitlist groups. The resulting total was (40x8) 320 participants.

#### Data analysis

After data collection was completed, all paper-based files were transcribed to a spreadsheet database. The de-identified database was sent to the investigators for analysis.

Participants were stratified into *Type of participant group:* Adult, Adult + proxy, Child, and Child + proxy. Those who were not independently propelling the wheelchair were classified under the proxy groups. Age was calculated by subtracting the baseline date minus DOB and dividing by 365.

Descriptive statistics of demographics information, chi-square, and Fisher’s exact test were performed to compare for group (Waitlist and Wheelchair) differences at baseline. For each standardized questionnaire, subscores and total scores were calculated (CHART-SF, WHOQOL-BREF, FMA, FMA kids, WST-Q user, WST-Q caregiver, and WAC). Then, a score change was calculated for the measures that were collected at baseline and follow up (CHART-SF, WHOQOL-BREF, FMA, FMA-kids). Shapiro-Wilk test was performed to check for normal distribution. The data was not normally distributed. Therefore, the Mann–Whitney *U* Test was run to evaluate if there was a difference between questionnaire delta scores of participants who received a wheelchair and participants that were on the waiting list. Change in the absence or presence of pressure ulcers between baseline and follow up measures was coded as deterioration, no change, and improvement. Fisher exact test was run to evaluate if there was a difference between the Waitlist and the Wheelchair group stratified by whether they had a wheelchair at baseline or not. Descriptive statistics for WAC scores and WST-Q were also run and stratified by type of wheelchair provided. Wilcoxon signed rank test was used to assess if there was a difference in WST-Q scores between baseline and follow up for those who had a wheelchair at baseline in the Wheelchair group. Kruskal-Wallis was used to evaluate if there were differences in daily wheelchair use in hours and WAC score by type of wheelchair provided for each type of participant. The level of significance was set at *p* = 0.05. To control for type I error, post-hoc analysis p-value for the Kruskal-Wallis test was set at *p* = 0.008. Effect size was calculated for the results that were statistically significant $\left(r=\left|z\right|/\sqrt{n}\right)$. SPSS version 21 was used to perform all statistical analyses. Participants were excluded from the analysis if they passed away before the study conclusion.

## Results

A total of 344 participants were enrolled in the study. Of these, 29 passed away before the completion of the study and 6 were missing *Type of participant* information and were not included in the analysis. 167 participants were in the Waitlist group and 142 in the Wheelchair group. The average time between baseline and follow up was 193 ± 27 days. Descriptive statistics of age, gender, and having a wheelchair at baseline stratified by type of participant and group are presented in Table 2 and for disability type in Table 3. No significant differences between Waitlist and Wheelchair group were found for any type of participant at baseline for age (*p* > 0.309), gender (*p* > 0.229), number of participants with a wheelchair at baseline (*p* > 0.077), and disability type (*p* > 0.127). Of those who did not have a wheelchair at baseline and provided information on means of mobility (*n* = 210), 15 % reported ambulating with crutches/walkers/canes, 48 % crawled, and 37 % were carried by others. For the Wheelchair group, the types and frequencies of wheelchairs provided are listed on Table 4. There was a significant difference in average hours of wheelchair use by type of wheelchair provided for Adults (*p* = 0.049). However, the post-hoc comparisons did not result in any significant differences between wheelchairs.

**Table 2:** Demographics descriptive statistics for age, gender, and having a wheelchair at baseline

Participant (*n* = 309)		Age Mean ± SD (6 missing)		Gender		Had a wheelchair at baseline			
						Male (4 missing)		Female (6 missing)	
		M	F	M	F	Y	N	Y	N
A (*n* = 96, 31 %)	WL (*n* = 41, 43 %)	38.9 ± 12.8	34.7 ± 15.1	30, 73 %	11, 27 %	7, 23 %	22, 73 %	1, 9 %	9, 82 %
	WC (n = 55, 57 %)	41.6 ± 10.6	36.4 ± 14.5	37, 67 %	18, 33 %	13, 35 %	24, 65 %	8, 44 %	10, 56 %
A + p (*n* = 60, 19 %)	WL (*n* = 38, 63 %)	36.7 ± 18.8	35.6 ± 19.4	25, 66 %	13, 34 %	3, 12 %	21, 84 %	1, 8 %	12, 92 %
	WC (*n* = 22, 37 %)	23 ± 10.1	40.2 ± 21.8	11, 50 %	11, 50 %	1, 9 %	9, 82 %	1, 9 %	9, 82 %
C (*n* = 6, 2 %)	WL *n* = 0	0	0	0	0	0	0	0	0
	WC (*n* = 6, 100 %)	11.3 ± 3.3	7.4	5, 83 %	1, 17 %	0	5, 100 %	0	1, 100 %
C + p (*n* = 147, 48 %)	WL (*n* = 88, 60 %)	10.1 ± 4	10.7 ± 5.4	56, 64 %	32, 36 %	7, 13 %	49, 87 %	0	28, 88 %
	WC (*n* = 59, 40 %)	10.3 ± 3.7	10.4 ± 6.4	34, 58 %	25, 42 %	5, 15 %	28, 82 %	0	25, 100 %

*F* Female, *M* Male, *Y* yes, *N* no, *A* Adult, A + p: Adult + proxy, C: Child, C + p: Child + proxy, *WL* waitlist, *WC* wheelchair

**Table 3:** Disability type frequencies by type of participant

Type of participant	Group	Disability type									
		CP	Polio	SCI, paralysis	Stroke, brain injury	Down syndrome, intellectual	Amputation	Diabetes	MS, MD	Other	Missing
A	WL	7	7	18	0	0	6	0	1	2	0
	WC	10	18	18	0	1	5	2	0	1	0
A + p	WL	10	3	7	8	1	1	3	0	5	0
	WC	11	1	2	2	1	0	3	2	0	0
C	WL	0	0	0	0	0	0	0	0	0	0
	WC	3	1	1	0	0	0	0	0	1	0
C + p	WL	75	0	0	0	3	0	5	0	5	0
	WC	50	1	0	0	1	0	4	1	1	1

*A* Adult, A + p: Adult + proxy, *C* Child, C + p: child + proxy, *SCI* spinal cord injury, *MS* Muscular sclerosis, *MD* muscular dystrophy, *WL* waitlist, *WC* wheelchair

**Table 4:** Types of wheelchairs provided and self-reported average daily use in hours at follow up

Type of participant	Wheelchair type							
	Kids		Roughrider		Harmony		Specialized	
	n	Hrs/day (mean ± SD)	n	Hrs/day (mean ± SD)	n	Hrs/day (mean ± SD)	n	Hrs/day (mean ± SD)
A*	0	-	37	4.7 ± 3.6	17	6.4 ± 3.8	1	.5
A + p	6	2.5 ± 1.4	4	1.5 ± .6	11	3.5 ± 2.3	1	2
C	4	6.3 ± 1.4	2	3.5 ± 3.5	0	-	0	-
C + p	53	3.8 ± 2.9	4	2.6 ± 1.5	2	9 ± 4.2	0	-

A: Adult, A + p: Adult + proxy, C: Child, C + p: child + proxy

^*^ *p* = 0.04

At baseline, 279 participants reported not having a pressure sore, 23 reported having a pressure sore, and 7 had missing information. At follow up, 289 participants reported not having a pressure sore, 18 reported having a pressure sore and 2 had missing information. No significant differences were found in presence/absence of pressure sores between the waiting list and the wheelchair group for all types of participants, *p* > 0.276. Approximately 80 % of children were not enrolled in school and 60 % of adults were not employed at baseline and follow up. There was no significant difference in number of children enrolled in school or adults working between baseline and follow up for the Waitlist or the Wheelchair group.

In terms of participation measures using the CHART, Children with proxies in the Waitlist group reported a significant decrease in Mobility domain as compared to Children with proxies in the Wheelchair group, *p* = 0.023 (Table 5). Female Adults who received a wheelchair reported a larger increase in physical health than those females who were on the waiting list as measured by the WHOQOL-BREF (Table 6). Both male and female Adults who received a wheelchair also reported significantly better Environment health than those Adults on the waiting list as measured by the WHOQOL-BREF (Table 6).

**Table 5:** CHART domain change scores (follow up – baseline) by type of participant compared by waitlist and wheelchair group

Type of participant	CHART Domain M (IQR)											
	Physical		Cognitive		Mobility		Occupation		Social integration		Economic self sufficiency	
	WL	WC	WL	WC	WL	WC	WL	WC	WL	WC	WL	WC
Adults	0(0) *n* = 7	0(0) *n* = 45	0(22)	0(0)	0(14)	0(0)	0(0)	0(0)	C	0(3)	C	0(0)
Adults + proxy	C *n* = 12	0(0) *n* = 14	0(0)	0(12)	0(5.6)	0(2.6)	0(.47)	C	C	0(6.5)	C	0(0)
Child + proxy	0(0) *n* = 22	0(0) *n* = 21	0(0)	C	0(.75)^*^	0(0)^*^	0(0)	0(0)	0(0)	0(1.25)	C	0(0)

*M* median, *IQR* interquartile range, *C* constant, *WC* wheelchair, *WL* waitlist

^*^ *P* < 0.023, *r* = 0.22, β = 0.29

**Table 6:** WHO domain change scores stratified by gender

Type of participant	WHO domain Median (IQR)							
	Physical health		Psychological		Social relationships		Environment	
	WL	WC	WL	WC	WL	WC	WL	WC
A male	0(7.14) *n* = 26	0(3.57) *n* = 33	0(8.33)	0(4.17)	0(0)	0(13.54)	0(6.25)^**^	3.12(6.25)^**^
A female	0(3.57)^*^ *n* = 11	3.57(7.14)^*^ *n* = 12	0(4.17)	0(9.38)	0(0)	0(0)	0(3.13)^***^	3.12(5.47)^***^
A + p male	0(3.57) *n* = 12	1.78(8.93) *n* = 4	0(8.33)	constant	0(8.33)	0(9.38)	0(4.46)	1.56(3.13)
A + p female	0(7.14) *n* = 5	0(3.57) *n* = 7	0(6.25)	0(4.17)	constant	constant	0(1.56)	3.12(9.38)

*A* Adult, A + p: Adult + proxy, *C* Child, C + p: child + proxy, *WL* waitlist, *WC* wheelchair

^*^ *p* = 0.044, *r* = 0.43; β = 0.56, ^**^ *p* = 0.007, *r* = 0.35; β = 0.77, ^***^ *p* = 0.016, *r* = 0.53, β = 0.79

Satisfaction with the means of mobility based on the FMA was also significantly improved in Children with proxies (*p* < 0.001) and Adults with proxies (*p* = 0.021) who received a wheelchair as compared to the waiting list (Table 7). There was a trend in increased satisfaction with the means of mobility for Adults who received a wheelchair as compared to those on the waitlist (*p* = 0.073).

**Table 7:** FMA change scores for children (FMA kids) and adults (FMA)

Type of participant	n		Total score delta Median (IQR)	
	Waitlist	Wheelchair	Waitlist	Wheelchair
Adults	18	14	0(7.25)^***^	6(10)^***^
Adults + proxy	15	12	0(6)^**^	5.5(7.5)^**^
Child	0	6	-	5(5.75)
Child + proxy	67	52	0(1)^*^	5(6.75)^*^

^*^ *P* < 0.001, *r* = 0.50, β = 0.99; ^**^ *p* = 0.021, *r* = 0.44, β = 0.74; ^***^ *p* = 0.073

Descriptive statistics for WST-Q scores for the participants who received a wheelchair are listed in Table 8. 28 % of Adults who received a wheelchair reported been able to go over a step and 45 % reported being able to go down a step. Only 11 % (6/53) reported that they were able to perform and hold a “wheelie” as well as turn while holding one. All Children (*n* = 4) reported not being able to perform a wheelie. There were 2 missing answers for Children and for Adults. For participants in the Wheelchair group who had a wheelchair at baseline, no significant differences between baseline and follow up were found in WST-Q capacity and performance scores for Children + proxy (*n* = 5) and Adults (*n* = 7). Due to small sample size it did not compute for Adults + proxy (*n* = 2) and Children (*n* = 0). For participants in the wheelchair group, the total WAC scores are described in Table 9. WAC scores were significantly higher in roughriders than in kids wheelchairs for Children with proxies (*p* = 0.019). Few participants self-reported needing repairs. Casters needing repairs was reported by 2 Adults using roughriders and 3 Children with proxies using kids wheelchair. The seat needed a repair in 2 Adult roughriders. The peripherals (arms supports, push handles, etc.) needed repair in one harmony used by an Adult and one kids wheelchair used by a Child with a proxy. Two frames needed repair, one in an Adult harmony and one in a kids wheelchair. One foot support needed a repair in a harmony wheelchair. Two Adults, one using a roughrider and one using a harmony wheelchair, reported being stranded because of the wheelchair needing repair.

**Table 8:** Wheelchair skills scores for users and caregivers at follow up for those who were in the wheelchair group

WSTQ sub-score	Participant type mean ± SD			
	A n = 53	C *n* = 4	A + p *n* = 21	C + p *n* = 57
Capacity	70.6 ± 17.5	52.1 ± 19.35	77.7 ± 16.5	81.7 ± 16.18
Performance	66.1 ± 16.5	51.4 ± 20.3	76.6 ± 17.3	80.4 ± 15.6

*A* Adult, A + p: Adult + proxy, *C* Child, C + p: child + proxy

**Table 9:** WAC scores at follow up stratified by wheelchair type for those participants who received a wheelchair at baseline

Type of wheelchair	Type of participant							
	Adult	n	Adult + Proxy	n	Child	n	Child + Proxy	n
Roughrider	61.07(2.78)	36	61.6(.8)	4	61.02(.)	4	61.4(1.9)^*^	4
Kids	-	0	61.25(4.21)	6	59(4.50)	2	59.7(2.38)^*^	51
Harmony	61.9(4.10)	17	63(3)	11		0	56.28	1
Specialized	48.9	1	62.46	1		0		0

^*^ *p* = 0.019

## Discussion

Our results suggest that wheelchair provision according to the WHO 8-Steps had an overall positive impact on the life of the participants who received one as compared to those on the waiting list. To our knowledge, this is the first cohort study that examines how participation, quality of life, functional mobility, wheelchair skills, and wheelchair maintenance are affected by appropriate wheelchair provision in a less resourced setting. Surprising results included the few reported repairs needed in the past 6-months. As compared to an exploratory study in Mexico [32], this may indicate the positive impact of the better quality wheelchairs provided. Both the kids and the adult roughrider wheelchairs are reported to be compliant with the International Organization for Standardization (ISO) 7176 standards [63]. The wheelchairs in the Mexico study did not meet those standards [32]. This suggests that the threshold of meeting ISO 7176 standard as argued in the WHO Consensus meeting [37] would help improve reliability of wheelchairs. Studies in the US have reported that more than 40 % of wheelchair users with spinal cord injury have needed a repair in the past 6 months [64–66]. This discrepancy between the US data and the Indonesia data might be because the wheelchairs in Indonesia were only 6 months old at follow up. Further follow up could help elucidate the types of repairs inherent to the wheelchairs provided by UCPRUK.

In terms of demographics, 49 % (*n* = 153) of our sample were children, which is a step towards an increased understanding of the impact of wheelchair provision on this population. Other studies that have included children have had 20 % of the total sample at most and have had small sample sizes (maximum of 60) [24, 30, 31, 41, 45]. 80 % of the study participants did not have a wheelchair at baseline which is similar to baseline data from studies in India, Peru, Vietnam, and Chile with more than 90 % of their participants [30, 31]. Average hours of wheelchair use was higher in this study compared to those reported in studies that have provided a single-size wheelchair to all of the users [23, 30, 31]. However, wheelchairs provided in this study were not used on average more than a third of the day. This could be explained by the fact that school and employment status was not affected by access to a wheelchair. Barriers to participation such as accessibility, public transportation, and attitudinal barriers need to be investigated further.

The only significant difference in CHART domain scores was in the Mobility domain in Children with proxies. Children with proxies in the Waitlist group reported a decrease in Mobility and the Children with proxies in the Wheelchair group did not. This might be explained by the fact that as children grow older it is more difficult for their caregivers to carry them. Therefore, they may spend more time in bed or at home. This also suggests that even with a wheelchair provided through the WHO 8-Steps, improvement in mobility is not guaranteed; more investigations into factors that continue to limit mobility are necessary.

The environmental health domain in the WHOQOL-BREF was improved for both men and women who received a wheelchair. This domain covers issues related to financial resources, safety, health and social services, living physical environment, opportunities to acquire new skills and knowledge, recreation, general environment and transportation [56].

Children with proxies and Adults with proxies who received a wheelchair reported improved satisfaction with their mobility means as compared to those on the waiting list. A surprising result was that there was no significant difference in Adults in satisfaction with current mobility device as measured by the FMA between those in the Waitlist group and those in the Wheelchair group. However, it might be explained by the low scores in wheelchair skills. Training in wheelchair skills has been found to increase the likelihood for reporting improvements in satisfaction [25]. The WST-Q scores are comparable to the objective wheelchair skills test scores [58, 59]. The WST-Q scores of the Adults in this study at follow up are similar to the pre-training scores of adults in previous randomized control trials [67, 68]. None of the Child users and only 11 % of Adult users reported being able to perform a wheelie. This skill is essential to perform the most advanced skills [69] which are instrumental to navigate through architectural barriers often found in less resourced settings [1, 11, 20, 22, 33, 41, 70]. In addition, Caregiver scores for Adults with proxiesand Children with proxies were similar to pre-training scores in the wheelchair skills training study for caregivers [71]. This suggests that caregivers could also benefit from more wheelchair skills training. Even though the WHO WSTP provided by UCPRUK includes training the user in mobility skills, only seven basic mobility skills are taught and it does not include the wheelie [38]. These results may suggest the need for further mobility skills training of wheelchair personnel that could then translate to training of wheelchair users. Several wheelchair skills training programs exist including the Wheelchair Skills Program from Dalhousie University in Canada [68, 71, 72] and the boot camp offered by the Vida Independiente organization in Mexico city [73].

Our study yielded mixed results that call for more rigorous study of the impact of wheelchair provision in a less-resourced setting.

### Limitations

The Child group had a very small sample size (*n* = 6). Since participants were recruited as they came in for services on first-come first-served basis, additional efforts to recruit more for this group could not be made in the time allotted for the study. In addition, characteristics of wheelchair users in Indonesia are unknown; therefore we cannot explain whether most of the children who require a wheelchair are not independent for manual wheelchair mobility. Although the number of participants in the Waitlist group and the Wheelchair group was close to the goal in the power analysis, there was a considerable amount of missing data for the questionnaires. Even though all questionnaires were translated to Bahasa Indonesia, back translated to English and re-revised during the data collectors training, the reliability of the translated questionnaires is unknown. A reason for the missing data could be that some questions were not clear to participants. The WHOQOL-BREF questionnaire, a validated tool, had the least amount of missing data for the Adult group. However, some of the questionnaires have been used successfully in other languages and contexts, such as the WST-Q [74] and CHART-SF [75, 76] . There is a significant need for the development or implementation of a rigorous assistive technology or wheelchair outcome measure that is validated across cultures and languages [77]. A start may be the validation of the FMA in different languages and contexts, which could aid to collect datasets at different sites and gather them to help guide policy. Self-reported data might not correlate to objective data and responses may be given to satisfy the interviewer, as encountered in a previous study [1]. Another potential explanation for the missing data is that both the participant and the data-collector were bored. The protocol lasted around 2 h and was entirely paper-based. Questions may have been missed. An additional limitation to this data collection measure was that data was digitized only after all of it was collected. It is likely that entry errors occurred; we could not control for that.

This was a convenience sample, and thus not a randomly selected sample. Therefore, there is a risk for selection bias. Furthermore, this study was funded as part of UCP’s grant to provide wheelchair services in Indonesia. The study was designed and the data was analyzed by an independent group of researchers, but the data was collected by a team from UCP, opening up a small potential for bias.

We only studied a cohort of users that had received the services based on the WHO 8-Steps; future studies should consider a control group receiving wheelchairs through different delivery mechanisms to compare and contrast differences with service delivery methods.

Future work to address these limitations should also include studying the cost-effectiveness of the service provision model [15, 77].

## Conclusion

This study found that provision of wheelchairs fitted according to the WHO 8-Step approach in Indonesia resulted in greater satisfaction with mobility for Children with proxies and Adults with proxies. Additionally, Adults that received wheelchairs reported a better quality of life than those on the waiting list. These results indicate that wheelchair provision in low-income countries has a range of positive effects on adults, but could be improved to better satisfy the rehabilitation needs of children. Wheelchair skills were found to be a confounding factor which could influence mobility measurements, and thus additional effort should be focused on providing wheelchair skills training.

## Abbreviations

AT: assistive technology
CHART-SF: Craig Handicap Assessment Recording Technique Short Form
ISO: International Organization for Standardization
Pitt: University of Pittsburgh
UCP: United Cerebral Palsy
UCPRUK: UCP Roda Untuk Kemanusiaan
UNCRPD: United Nations Convention on Rights of Persons with Disabilities
US: United States
USAID: Unites States Agency for International Development
WHO: World Health Organization
WHOQOL-BREF: World Health Organization Quality of Life-BREF
WHO WSTP: World Health Organization Wheelchair Service Training Package
WST-Q: Wheelchair Skills Test Questionnaire
WAC: Wheelchair Assessment Checklist
FMA: Functional Mobility Assessment

## References

[1] Borg J, Ostergren P-O, Larsson S, Rahman AA, Bari N, Khan AN (2012). Assistive technology use is associated with reduced capability poverty: a cross-sectional study in Bangladesh. Disabil Rehabil Assist Technol.

[2] Nations U (2006). Convention on the rights of persons with disabilities and optional protocol.

[3] Skempes D, Stucki G, Bickenbach J (2015). Health related rehabilitation and human rights: Analyzing States’ obligations under the United Nations Convention on the Rights of Persons with Disabilities. Arch Phys Med Rehabil.

[4] Scherer MJ, Sax C, Vanbiervliet A, Cushman L, Scherer J (2005). Predictors of assistive technology use: the importance of personal and psychosocial factors. Disabil Rehabil.

[5] Scherer M, Jutai J, Fuhrer M, Demers L, Deruyter F (2007). A framework for modelling the selection of assistive technology devices (ATDs). Disabil. Rehabil. Assist. Technol..

[6] Lenker JA, Paquet VL (2003). A review of conceptual models for assistive technology outcomes research and practice. Assist Technol.

[7] World Health Organization (2013). The International Spinal Cord Society. International Perspectives on Spinal Cord Injury.

[8] Ripat JD, Woodgate RL (2012). The role of assistive technology in self-perceived participation. Int J Rehabil Res.

[9] World Health Organization (2008). Guidelines on the provision of manual wheelchairs in less resourced settings.

[10] World Health Organization (2008). Guidelines on the provision of manual wheelchairs in less resourced settings.

[11] Borg J, Larsson S, Östergren PO (2011). The right to assistive technology: for whom, for what, and by whom?. Disability Society.

[12] World Health Organization (2011). World report on disability.

[13] Adya M, Samant D, Scherer MJ, Killeen M, Morris MW (2012). Assistive/rehabilitation technology, disability, and service delivery models. Cogn Process.

[14] Winter AG. Assessment of wheelchair technology in Tanzania. International Journal for Service Learning in Engineering, Humanitarian Engineering and Social Entrepreneurship. 2006;2(1):60-77.

[15] Borg J, Östergren P-O (2015). Users’ perspectives on the provision of assistive technologies in Bangladesh: awareness, providers, costs and barriers. Disabil. Rehabil. Assist. Technol..

[16] Hotchkiss R. Independence through mobility: a guide through the manufacture of the AT-Hotchkiss wheelchair. Washington D.C.: Appropriate Technology International; 1985.

[17] Motivation. Motivation Freedom Through Mobility. 2014. http://www.motivation.org.uk/. Accessed November 3, 2014 2014.

[18] Pearlman J, Cooper R, Zipfel E, Cooper R, McCartney M (2006). Towards the development of an effective technology transfer model of wheelchairs to developing countries. Disabil. Rehabil. Assist. Technol..

[19] Krizack M, Heinicke-Motsch K, Sygall S (2003). It’s not about wheelchairs. Building an inclusive development community: A manual on including people with disabilities in international development programs.

[20] Mines R (2008). Wheelchair Postural Support Solutions for Low-Income Countries. Ergon. Des..

[21] Armstrong W, Reisinger KD, Smith WK (2007). Evaluation of CIR-Whirlwind Wheelchair and service provision in Afghanistan. Disabil Rehabil.

[22] Pearlman J, Cooper R, Krizack M, Lindsley A, Wu Y, Reisinger K (2008). Lower-Limb Protheses and wheelchairs in low income countries: An overview. IEEE-EMBS Magazine.

[23] Mukherjee G, Samanta A (2005). Wheelchair charity: a useless benevolence in community-based rehabilitation. Disabil Rehabil.

[24] Toro ML, Garcia-Mendez Y, Dausey D, Pearlman J, editors. Comparison of a manual wheelchair designed and produced in Mexico to a wheelchair produced in China based on ISO testing and clinician and user feedback. RESNA Annual Conference; 2012; Baltimore, MD.

[25] Borg J, Larsson S, Östergren P-O, Rahman AA, Bari N, Khan AN (2012). User involvement in service delivery predicts outcomes of assistive technology use: A cross-sectional study in Bangladesh. BMC Health Serv Res.

[26] Guimaraes E, Mann WC (2003). Evaluation of pressure and durability of a low-cost wheelchair cushion designed for developing countries. Int J Rehabil Res.

[27] World Health Organization, United States Agency for International Development. Joint position paper on the provision of mobility devices in less resourced settings 2011.

[28] Phillips B, Zhao H (1993). Predictors of assistive technology abandonment. Assist Technol.

[29] Kim J, Mulholland SJ (1999). Seating/wheelchair technology in the developing world: need for a closer look. Technol Disabil.

[30] Shore S (2008). Use of an economical wheelchair in India and Peru: impact on health and function. Med Sci Monit.

[31] Shore S, Juillerat S (2012). The impact of a low cost wheelchair on the quality of life of the disabled in the developing world. Med. Sci. Monit..

[32] Toro ML, Garcia Y, Ojeda AM, Dausey DJ, Pearlman J (2012). Quantitative exploratory evaluation of the frequency, causes and consequences of rehabilitation wheelchair breakdowns delivered at a paediatric clinic in Mexico. Disability, CBR and Inclusive Development.

[33] Howitt J. Donated wheelchairs in low-income countries - issues and alternative methods for improving wheelchair provision. The 4th Institution of Engineering and Technology Seminar on Appropriate Healthcare Technologies for Developing Countries; 23–24 May: IEEE Explore; 2006. p. 39–44.

[34] Oderud T, editor. User satisfaction survey: an assessment study on wheelchairs in Tanzania. Report of a consensus conference on wheelchairs for developing countries, Bengaluru, India 6-11 November 2006: WHO, ISPO, and USAID; 2007.

[35] Hotchkiss R. Putting the tools in the hands that can use them: Wheelchairs in the Third World. RESNA 10th Annual Conference; San Jose, CA1987.

[36] Eide AH, Oderud T, MacLachlan M, Swartz L (2009). Assistive technology in low-income countries. Disability and international development: towards inclusive global health.

[37] Sheldon S, Jacobs NA. Report of a Consensus Conference on Wheelchairs for Developing Countries. Bengaluru, India 6-11 November 2006: WHO, ISPO, and USAID; 2007.

[38] World Health Organization. Wheelchair Service Training Package - Basic Level. 2012. http://www.who.int/disabilities/technology/wheelchairpackage/en/. Accessed October 24 2014.

[39] World Health Organization. Wheelchair Service Training Package - Intermediate Level. Geneva. 2013. http://www.who.int/disabilities/technology/wheelchairpackage/wstpintermediate/en/. Accessed November 3 2014.

[40] Constantine D, Hingley C, Jowitt J. Donated wheelchairs in low-income countries - issues and alternative methods for improving wheelchair provision. The 4th Institution of Engineering and Technology Seminar on Appropriate Healthcare Technologies for Developing Countries; 23–24 May. London, UK: IEEE Explore; 2006.

[41] Glumac LK, Pennington SL, Sweeney JK, Leavitt RL (2009). Guatemalan caregivers’ perceptions of receiving and using wheelchairs donated for their children. Pediatr Phys Ther.

[42] McPherson B. Hearing assistive technologies in developing countries: background, achievements and challenges. Disability and Rehabilitation: Assistive Technology. 2014(0):1–5. 10.3109/17483107.2014.907365 24702607

[43] Carkeet D, Pither D, Anderson M (2014). Developing self-sustainable hearing centers in the developing world-case study of EARs Inc project in Dominican Republic. Disabil. Rehabil. Assist. Technol..

[44] Ikeda AJ, Grabowski AM, Lindsley A, Sadeghi-Demneh E, Reisinger KD. A scoping literature review of the provision of orthoses and prostheses in resource-limited environments 2000–2010. Part one: considerations for success. Prosthetics and orthotics international. 2013:0309364613500690. 10.1177/0309364613500690 24026045

[45] Rispin K, Wee J (2013). A paired outcomes study comparing two pediatric wheelchairs for low resource settings; the Regency pediatric wheelchair and a similarly sized wheelchair made in Kenya. Assist Technol.

[46] Jefferds AN, Beyene NM, Upadhyay N, Shoker P, Pearlman JL, Cooper RA (2010). Current state of mobility technology provision in less-resourced countries. Phys. Med. Rehabil. Clin. N. Am..

[47] Pearlman J, editor. Review session: Review of literature on wheelchairs for developing countries & Review of wheelchair provision in developing countries. Consensus Conference on Wheelchairs for Developing Countries; 2006.

[48] Borg J, Lindstrom A, Larsson S (2009). Assistive technology in developing countries: national and international responsibilities to implement the Convention on the Rights of Persons with Disabilities. Lancet.

[49] Borg J, Lindstrom A, Larsson S (2011). Assistive technology in developing countries: a review from the perspective of the Convention on the Rights of Persons with Disabilities. Prosthetics Orthot Int.

[50] Wheels for Humanity Indonesia. UCP Roda Untuk Kemanusiaan. 2014. http://ucpruk.org/about-us/about-upruk/. Accessed October 20 2014.

[51] Filmer D (2008). Disability, poverty, and schooling in developing countries: results from 14 household surveys. World Bank Econ. Rev..

[52] Kusumastuti P, Pradanasari R, Ratnawati A (2014). The Problems of People with Disability in Indonesia and What Is Being Learned from the World Report on Disability. Am. J. Phys. Med. Rehabil..

[53] Carson MC, editor. Advancement of appropriate rehabilitation technology in Indonesia. RESNA Annual Conference; 1994; Nashville, TN: RESNA Press.

[54] Whiteneck G, Brooks CA, Charlifue S, Gerhart KA, Mellick D, Overholser D (1992). Guide for use of the CHART: Craig Handicap Assessment and Reporting Technique.

[55] Hall KM, Dijkers M, Whiteneck G, Brooks C, Stuart KJ (1998). The Craig handicap assessment and reporting technique (CHART): metric properties and scoring. Topics Spinal Cord Injury Rehabil.

[56] World Health Organization (1998). Development of the World Health Organization WHOQOL-BREF quality of life assessment. The WHOQOL Group. Psychol Med.

[57] Kirby RL. Wheelchair Skills Program Version 4.1 Wheelchair Skills Test Manual. In: University D, editor.2011.

[58] Mountain A, Kirby RL, Smith C (2004). The wheelchair skills test, version 2.4: validity of an algorithm-based questionnaire version. Arch Phys Med Rehabil.

[59] Rushton PW, Kirby RL, Miller WC (2012). Manual wheelchair skills: objective testing versus subjective questionnaire. Arch Phys Med Rehabil.

[60] Karmarkar A, Collins DM, Cooksley C. Development of a Wheelchair Assessment Checklist: Preliminary Psychometric Analysis Annual RESNA Conference; New Orleans, LA2009.

[61] Kumar A, Schmeler MR, Karmarkar AM, Collins DM, Cooper R, Cooper RA (2013). Test-retest reliability of the functional mobility assessment (FMA): a pilot study. Disabil Rehabil Assist Technol.

[62] Rispin K, Schein R, Wee J. A Modification of the Functional Mobility Assessment for use with School Children in Kenya. 29th International Seating Symposium; Nashville, TN2013.

[63] UCP Wheels For Humanity. Wheelchair Provision. 2014. http://ucpruk.org/programs-services/wheelchair-provision/. Accessed November 15 2014.

[64] Worobey L, Oyster M, Nemunaitis G, Cooper R, Boninger ML (2012). Increases in wheelchair breakdowns, repairs, and adverse consequences for people with traumatic spinal cord injury. Am J Phys Med Rehabil.

[65] McClure L, Boninger M, Oyster M, Williams S, Houlihan B, Lieberman J (2009). Wheelchair repairs, breakdown, and adverse consequences for people with traumatic spinal cord injury. Arch Phys Med Rehabil.

[66] Toro ML, Pearlman J, Oyster M, Boninger M, editors. Type and Frequency of Reported Wheelchair Repairs and Adverse Consequences Among People with Spinal Cord Injury. Rehabilitation Engineering and Assistive Technology Society of North America Conference; 2014 June 11–15; Indianapolis.

[67] MacPhee AH, Kirby RL, Coolen AL, Smith C, MacLeod DA, Dupuis DJ (2004). Wheelchair skills training program: A randomized clinical trial of wheelchair users undergoing initial rehabilitation. Arch Phys Med Rehabil.

[68] Best KL, Kirby RL, Smith C, MacLeod DA (2005). Wheelchair skills training for community-based manual wheelchair users: a randomized controlled trial. Arch Phys Med Rehabil.

[69] Kirby RL, Kirby RL, Smith C, Seaman R, Macleod DA, Parker K (2006). The manual wheelchair wheelie: A review of our current understanding of an important motor skill. Disabil. Rehabil. Assist. Technol..

[70] Kirby RL, Kirby RL, Cooper RA (2007). Applicability of the Wheelchair Skills Program to the Indian context. Disabil. Rehabil..

[71] Kirby RL, Mifflen NJ, Thibault DL, Smith C, Best KL, Thompson KJ (2004). The manual wheelchair-handling skills of caregivers and the effect of training. Arch Phys Med Rehabil.

[72] Dalhousie University. Wheelchair Skills Program. 2012. http://www.wheelchairskillsprogram.ca/eng/index.php. Accessed November 3 2014.

[73] Vida Independiente Mexico. Vida Independiente Mexico. 2014. http://vidaindependientemexico.com/?page_id=498. Accessed November 3 2014.

[74] Routhier F, Kirby RL, Smith C, Demers L. The wheelchair skills program: relevance to the European setting. vol 20: Challenges for Assistive Technology. 2007.

[75] Tozato F, Tobimatsu Y, Wang C-W, Iwaya T, Kumamoto K, Ushiyama T (2005). Reliability and validity of the Craig Handicap Assessment and Reporting Technique for Japanese individuals with spinal cord injury. Tohoku J Exp Med.

[76] Golhasani-Keshtan F, Ebrahimzadeh MH, Fattahi AS, Soltani-Moghaddas SH, Omidi-Kashani F (2013). Validation and cross-cultural adaptation of the Persian version of Craig Handicap Assessment and Reporting Technique (CHART) short form. Disabil Rehabil.

[77] Bray N, Noyes J, Edwards RT, Harris N (2014). Wheelchair interventions, services and provision for disabled children: a mixed-method systematic review and conceptual framework. BMC Health Serv Res.

